# Reconstruction of a malignant soft tissue tumor around the elbow joint using a frozen autograft treated with liquid nitrogen, in combination with a free anterolateral thigh flap: A report of two cases

**DOI:** 10.1080/23320885.2018.1539911

**Published:** 2018-11-08

**Authors:** Akihiro Hirakawa, Akihito Nagano, Shingo Komura, Daichi Ishimaru, Kenji Kawashima, Haruhiko Akiyama

**Affiliations:** Department of Orthopaedic Surgery, Gifu University School of Medicine, Gifu, Japan

**Keywords:** elbow, malignant soft tissue tumor, frozen autograft, free anterolateral thigh flap

## Abstract

We present two cases of malignant soft tissue tumor around the elbow joint treated with *en bloc* resection, in which reconstruction using a frozen autograft technique in combination with a free anterolateral thigh flap offered reliable method for limb salvage and good recovery of elbow function.

## Introduction

Biological reconstruction is a useful procedure for limb salvage surgery after wide resection of malignant bone and soft tissue tumors. In particular, reconstruction using a frozen autograft treated with liquid nitrogen is a simple and effective method [1,2]. Additionally, oncosurgical wide resection of distally located soft tissue sarcoma often requires soft tissue reconstruction, including the use of a free tissue transfer [3]. To the best of our knowledge, there have been no previous reports of a reconstruction using a frozen autograft, treated with liquid nitrogen, around the elbow region; nor are there previous reports of reconstruction using a frozen autograft technique in combination with a free flap technique. Our aim in this report was to describe two cases of malignant soft tissue tumor around the elbow joint reconstructed using a combination of frozen autograft and a free anterolateral thigh (ALT) flap.

All procedures followed were in accordance with the ethical standards of the responsible committee on human experimentation (institutional and national) and with the Helsinki Declaration of 1975, as revised in 2008. Informed consent was obtained from all patients to be included in the study.

## Case presentation

### Case 1

**Figure 1.**
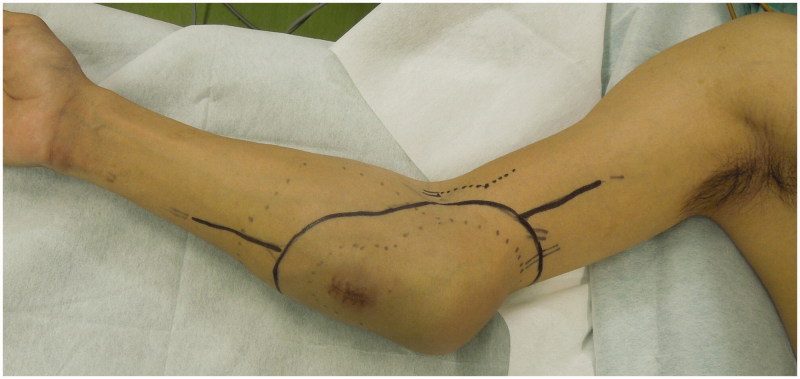
Pre-operative clinical photograph of the 41-year old man with a synovial sarcoma in the right medial elbow region.

**Figure 2.**
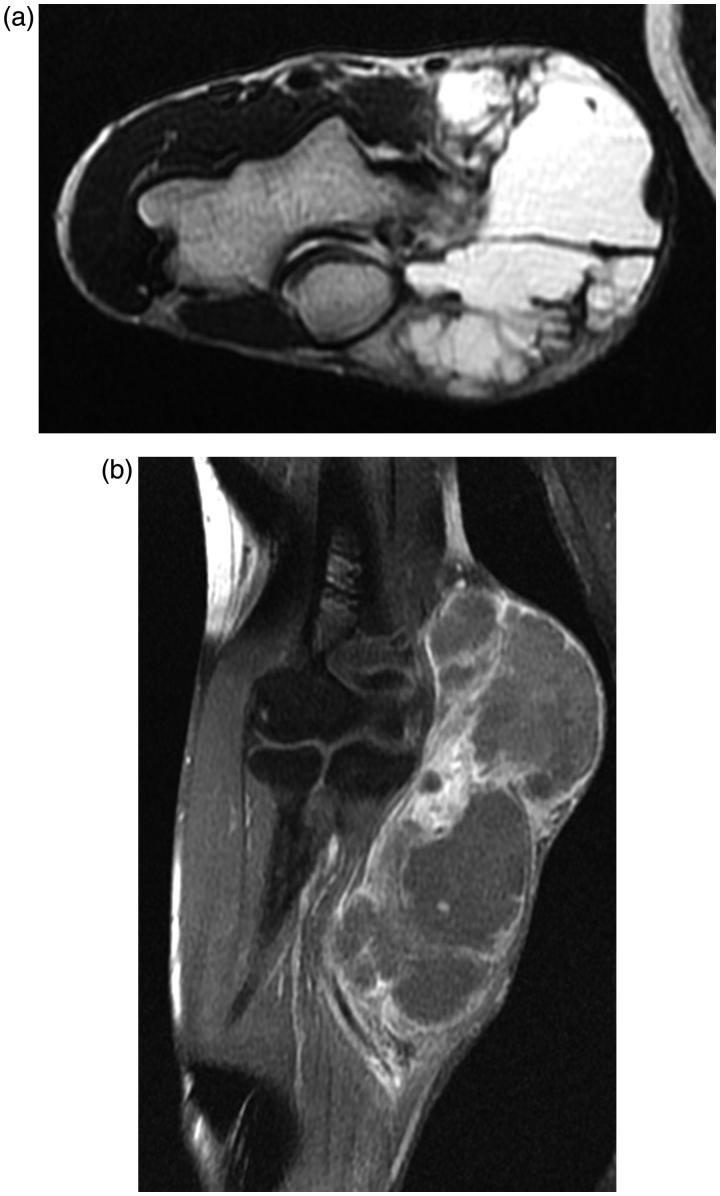
Pre-operative magnetic resonance image, showing the axial T2WI (a), and coronal Gd-DTPA-enhanced T1WI (b).

**Figure 3.**
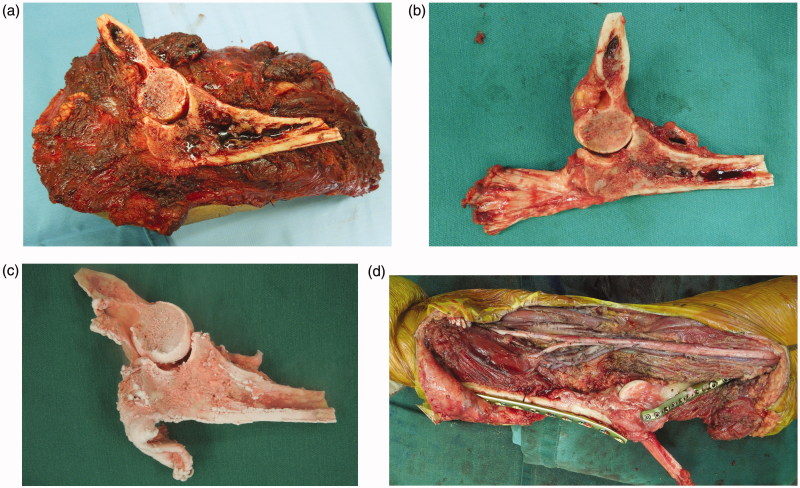
Intraoperative photographs showing the frozen autograft technique. The tumor was excised *en bloc* with a wide (2 cm) margin (a). All soft tissue and the tumor were dissected from the bone, with exception of the articular capsule and the tendon of triceps brachii with its insertion (b). The bone specimen was treated in liquid nitrogen (c). The frozen autograft was fixed *in situ* with plates (d).

A 41-year-old man was referred to our hospital with a two-year history of a tumor in his right elbow. Physical examination confirmed a 75 × 51 × 15 cm mass localized on the medial aspect of the right elbow (Figure 1). The range of motion (ROM) of the elbow was within normal limits. Plain radiographs revealed the shadow of a soft tissue mass, with no abnormal findings in the humerus, radius, and ulna. On T1-weighted magnetic resonance (MR) imaging (T1WI), the lesion showed mainly as an iso intensity. On T2-weighted MR imaging (T2WI) (Figure 2a), the lesion presented as a high intensity. On gadolinium-diethylenetriamine penta-acetic acid (Gd-DTPA)-enhanced T1WI, the lesion presented an inhomogeneous contrast enhancement (Figure 2b). Subsequent pathological examination of biopsy tissue confirmed a diagnosis of synovial sarcoma. After three courses of neoadjuvant chemotherapy, a wide tumor excision, with a 2-cm safety margin, defined on the basis of a brightness change of Gd-DTPA-enhanced T1WI, was performed, followed by reconstruction using an autograft treated with liquid nitrogen and a free ALT flap. The tumor was excised *en bloc* (Figure 3a), with the level of resection determined based on preoperative MR images. The following muscles were included in the resection: pronator teres, wrist and finger flexors, brachialis, anconeus, and part of the triceps brachii. The ulnar nerve was sacrificed out of necessity, but the median nerve was preserved by using ethanol as an adjuvant [4]. One third of the medial humerus and ulna were also resected using a bone saw. With the exception of the articular capsule attached to the humerus and the tendon of the triceps brachii with its insertion, all other soft tissues and the tumor were dissected from the bone sections (Figure 3b). The resected bone was then frozen in liquid nitrogen for 20 min, thawed in air at room temperature for 15 min and thawed in distilled water for 10 min, according to a previously published protocol (Figure 3c). The bones were then reconstructed in situ using locking plates (LCP Distal Humerus Plate, LCP Metaphyseal Plate 3.5: DePuy Synthes, Zuchwil, Switzerland), a headless compression screw (4.5 mm HCS: DePuy Synthes) and a cannulated cancellous screw (3.0 mm CCS: MEIRA, Japan). The tendon of the triceps brachii was repaired using a triclosan-coated polidioxanone suture (PDS^®^ PLUS: Ethicon Inc., Somerville, NJ) (Figure 3d). An ALT musculocutaneous flap with a vastus lateralis muscle of appropriate size was harvested and the soft tissue defect at the elbow reconstructed. Arterial revascularization was performed end-to-end to the transected ulnar artery. Venous anastomosis was done end-to-end to the cephalic vein. The affected limb was elevated postoperatively and the elbow was immobilized for 14 days to obtain wound healing and to avoid the risk of flap failure. Subsequently, range of motion (ROM) exercise was initiated. Bone union was defined as trabecular bone continuity, which can be seen as filling of the host-graft junction gap [1]. Filling of the host-graft junction gap was observed 12 months after the operation.

**Figure 4.**
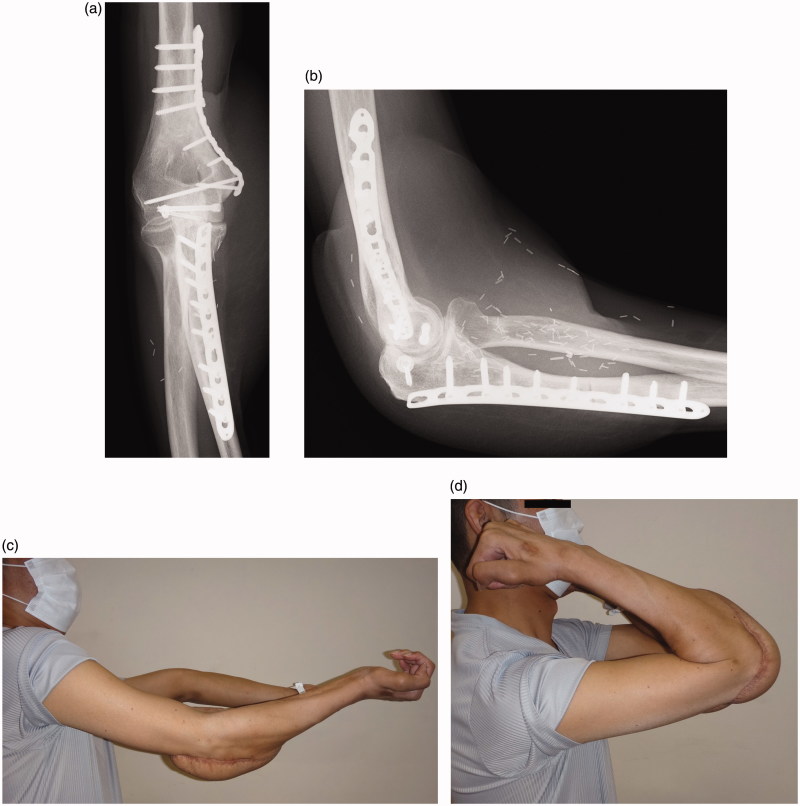
Follow-up radiograph and photograph obtained 2 years after the surgery. Anterior-posterior (a) and lateral (b) radiographs showing bony union of the osteotomy site, with no evidence of osteoarthritic changes at the elbow joint. Clinical photograph showing functional results in extension (c) and flexion (d).

At the 2-year follow-up, elbow ROM was −35° of extension and 130° flexion, and bone union was achieved (Figure 4a,b), with complete and stable coverage of the defect (Figure 4c,d). No local recurrence of the tumor was observed.

### Case 2

**Figure 5.**
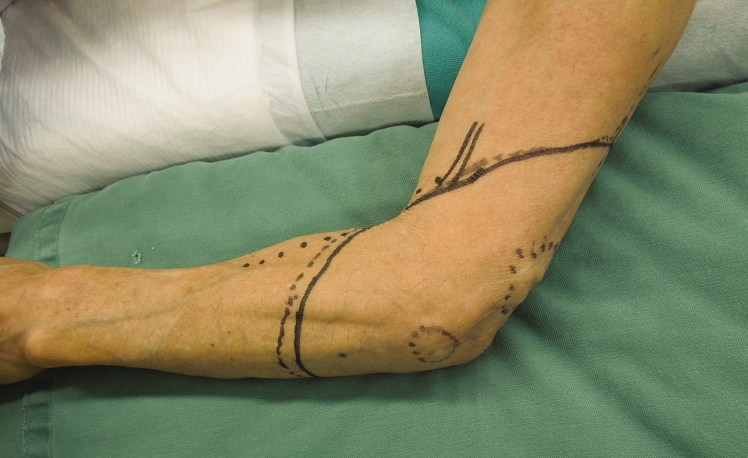
Pre-operative clinical photograph of the 73-year old woman, with a fibrosarcoma on the left lateral aspect of her elbow.

**Figure 6.**
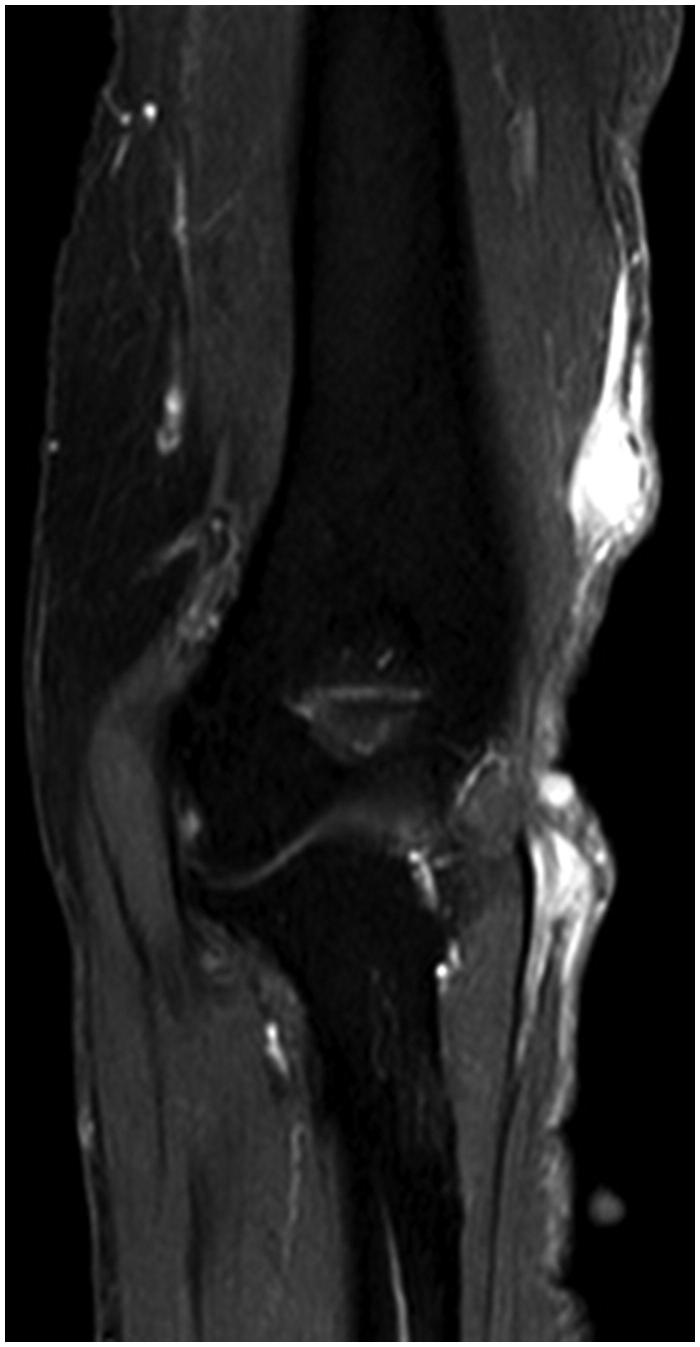
Pre-operative magnetic resonance image, showing the coronal Gd-DTPA-enhanced T1WI.

**Figure 7.**
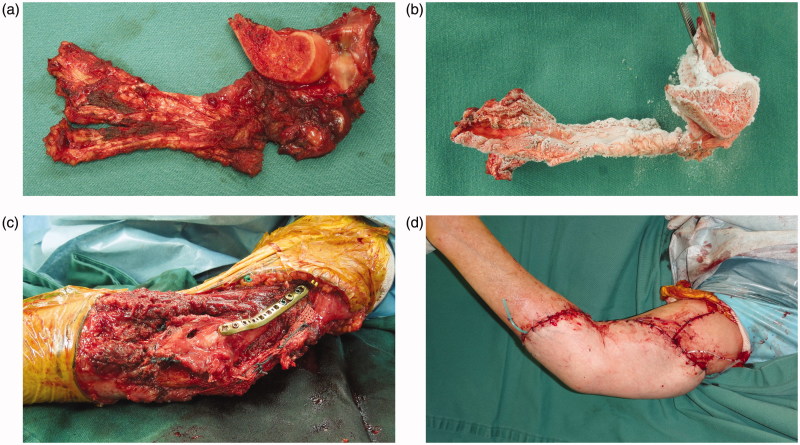
Intraoperative photographs showing the reconstruction, combining the frozen autograft technique with a free anterolateral thigh (ALT) flap. The tumor was excised *en bloc* with a wide (2 cm) margin (a). The resected specimen was treated in liquid nitrogen (b). The frozen autograft was fixed *in situ* with plates and a headless compression screw. Repair of the tendon of the triceps brachii and the radial articular capsule were repaired (c). The appropriately sized ALT (27 × 18 cm) flap was harvested and the soft tissue defect reconstructed (d).

**Figure 8.**
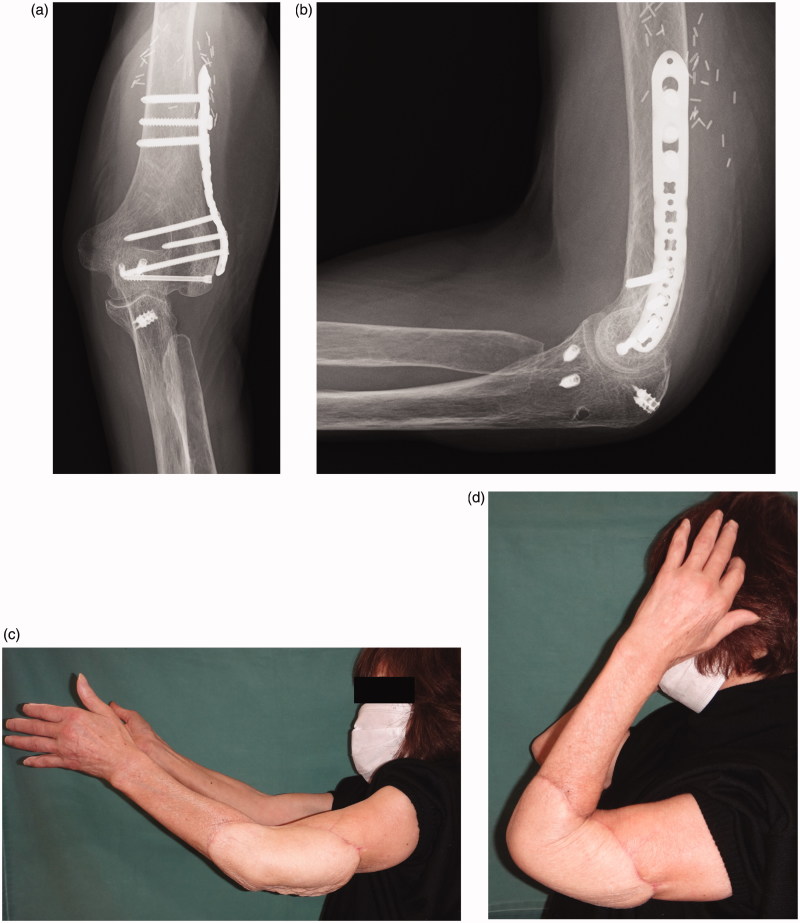
Follow-up radiograph and photograph obtained 12 months after surgery. Anterior-posterior (a) and lateral (b) radiographs showing bony union of the osteotomy site, with no evidence of osteoarthritic changes at the elbow joint. Clinical photograph showing functional results in extension (c) and flexion (d).

A 76-year-old woman visited her previous doctor with a six-month history of two tumors in her elbow. An excision biopsy was performed, with a diagnosis of fibrosarcoma confirmed through pathological examination. The patient was referred to our hospital for further assessment and treatment. On physical examination, two masses (2 × 2 cm in size) were identified on the lateral aspect of the right elbow (Figure 5), with the scar of a previous surgery between the two masses. The ROM of the elbow was within normal limits. No abnormal findings were identified on plain radiographs. On Gd-DTPA-enhanced T1WI, contrast enhancement was observed in the tumors and the fascia and subcutaneous tissue surrounding the lesions, which was considered as residual tumor tissue (Figure 6). The location of the skin incision and the level of resection were defined in the same manner as in Case 1. The tumor was excised *en bloc* (Figure 7a). Briefly, the wrist and finger extensor, supinator, anconeus, and triceps brachii were resected. One third of the lateral portion of the distal humerus and radial head were also resected using a bone saw. With the exception of the radial articular capsule attached to the humerus, the fascia of the wrist and finger extensor with its insertion and the tendon of the triceps brachii with its insertion, all other soft tissues and the tumor were dissected from the bone sections. The excised bone portion was frozen in liquid nitrogen in the same manner as in Case 1 (Figure 7b) and then reconstructed in situ using a locking plate (LCP Distal Humerus Plate: DePuy Synthes) and headless compression screw (3.5 mm HCS: DePuy Synthes). The triceps brachii, augmented with the Leeds-Keio ligament, was reattached to the olecranon and the radial articular capsule was reattached to the radial notch using a suture anchor (Corkscrew, Mini Corkscrew: Arthrex, Naples, FL). The wrist and finger extensor were repaired by using a polyethylene terephthalate suture (ETHIBOND^®^: Ethicon Inc.) (Figure 7c). The soft tissue defect was reconstructed with a free, 27 × 18 cm, ALT flap (Figure 7d). Arterial revascularization was performed end-to-end to the deep brachial artery. Venous anastomosis was done end-to-end to the vena comitans of the deep brachial artery and the basilic vein. The affected limb was elevated postoperatively and the elbow was immobilized for 14 days. Subsequently, ROM exercise was initiated in the same manner as in Case 1. Filling of the host-graft junction gap was observed 7 months after the operation. At the 1-year follow-up, elbow ROM was −35° extension and 130° flexion. Bone union was achieved (Figure 8a,b), and a complete and stable coverage of the defect was obtained (Figure 8c,d). Local recurrence of the tumor was not detected.

## Discussion

The goal of primary tissue reconstruction after sarcoma resection is to reconstruct the excised tissue and secure wound healing by filling the dead space [5]. In our two cases, for the reconstruction of excised bone, we used a frozen autograft technique, treating the autograft with liquid nitrogen, combined with a free ALT flap to correct the significant soft tissue defect.

Tsuchiya and colleagues [1,2] developed the frozen auto graft technique for biological reconstruction of large bone defects following excision of a bone tumor, in which the resected bone is treated with liquid nitrogen; favorable clinical and oncological outcomes for the management of bone and soft tissue sarcomas were reported for this method. However, in most cases, the technique has been used for the lower extremity or pelvis. Biological reconstruction using a frozen autograft treated with liquid nitrogen around the elbow region has not previously been reported.

Treatment with liquid nitrogen provides several clinical advantages, including maintenance of the osteoconductive properties of bone, good fit between the graft and host bone, sufficient biomechanical strength, the preservation of the cartilage matrix, and easy attachment of tendons and ligament to bone [1,2,6]. In addition, treatment with liquid nitrogen preserves the tendons and ligament, including their insertion. Accordingly, in the two cases presented in this report, bony union at the osteotomy site was achieved. Furthermore, the tendon of the triceps brachii (both cases) and the complex of the radial ligament (Case 2) were preserved, including their point of insertion on the bone, and repaired by direct suture or using a suture anchor, without difficulty.

Degeneration of articular cartilage over time has been reported as a disadvantage of this method [7]. Igrashi et al. [2] suggested that tumors with extensive involvement of the articular surface should be managed with both a composite graft and a prosthesis. However, in contrast to the hip and knee joint, the elbow is not a load-bearing joint. Therefore, articular cartilage of the elbow joint may be better preserved after liquid nitrogen freezing compared to knee joint cartilage, and may not progress to osteoarthritic changes. In both cases, there were no obvious findings of osteoarthritis of the elbow joint on follow-up radiographs. In the management of malignant bone and soft tissue tumor around elbow joint, reconstruction using a frozen autograft treated with liquid nitrogen should, therefore, be considered as a viable treatment option.

Free microsurgical tissue transfer is an important reconstructive option, providing adequate coverage of the defect following radical tumor resection, as well as reducing the risk of wound complications and enabling fast recovery of function and form and, thereby, avoiding delays in initiating adjuvant therapy and rehabilitation [8]. The ALT flap provides a large amount of skin and subcutaneous tissue, with the vastus lateralis muscle in the flap, which can be used to obliterate dead space and combat infection [9]. In our cases, the free ALT flap was selected taking into consideration the size and location of the defect, and the presence of exposed prosthetic materials, tendon, and bone. Particularly in Case 1, a free ALT musculocutaneous flap with a vastus lateralis muscle was harvested to cover bulky hardware and obliterate dead space. Successful coverage allowed early mobilization, which led to good postoperative function of the elbow joint.

In summary, we report two cases of malignant soft tissue tumor around the elbow joint in which reconstruction using a frozen autograft technique, with the bone treated in liquid nitrogen, in combination with a free ALT flap offered reliable method for limb salvage and good recovery of elbow function. Careful attention to local tumor recurrence and osteoarthritic changes of the elbow joint is necessary. Long-term follow up of cases will provide more useful information of this procedure.
